# The role of ADC values within the normal-appearing brain in the prognosis of multiple sclerosis activity during interferon-β therapy in the 3-year follow-up: a preliminary report

**DOI:** 10.1038/s41598-020-69383-3

**Published:** 2020-07-30

**Authors:** Anna Zacharzewska-Gondek, Anna Pokryszko-Dragan, Sławomir Budrewicz, Marek Sąsiadek, Grzegorz Trybek, Joanna Bladowska

**Affiliations:** 1 Department of General and Interventional Radiology and Neuroradiology, Wroclaw Medical University, 213 Borowska Street, 50-556 Wroclaw, Poland; 2 Department and Clinic of Neurology, Wroclaw Medical University, 213 Borowska Street, 50-556 Wroclaw, Poland; 3 Department of Oral Surgery, Pomeranian Medical University, 72 Powstańców Wielkopolskich Street, 70-111 Szczecin, Poland

**Keywords:** Multiple sclerosis, Prognostic markers

## Abstract

Predictors of multiple sclerosis (MS) activity during disease-modifying treatment are being extensively investigated. The aim of this study was to assess the prognosis of NEDA (no evidence of disease activity) status during IFN-β (interferon-β) treatment, using apparent diffusion coefficient (ADC) measurements obtained at initial MRI (magnetic resonance imaging). In 87 MS patients treated with IFN-β, ADC values were calculated for 13 regions of normal-appearing white and grey matter (NAWM, NAGM) based on MRI performed with a 1.5 T magnet before (MS0, n = 45) or after one year of therapy (MS1, n = 42). Associations were evaluated between ADC, conventional MRI findings, demographic and clinical factors and NEDA status within the following 3 years using logistic, Cox and multinomial logistic regression models. NEDA rates in the MS0 group were 64.4%, 46.5% and 33.3% after the 1st, 2nd and 3rd year of treatment, respectively and in MS1 patients 71.4% and 48.7% for the periods 1st–2nd and 1st–3rd years of treatment, respectively. ADC values in the NAWM regions contributed to loss of NEDA and its clinical and radiological components, with a 1–3% increase in the risk of NEDA loss (p = 0.0001–0.0489) in both groups. ADC measurements may have an additional prognostic value with regard to NEDA status.

## Introduction

Multiple sclerosis (MS) is an inflammatory disease causing multifocal demyelination and axonopathy within the brain^1^. Immunomodulatory therapy is applied to reduce activity and to delay the progression of this disease^2,3^. The course of MS, natural and modified by treatment, shows substantial variability and it is crucial to identify the subjects with active disease and poor response to treatment at an early stage, in order to optimise the therapeutic strategy^2,4^. To date, variable demographic and clinical factors^5–9^ and conventional radiological measures^8–14^ were investigated as potential predictors of the course of MS with mixed results, thus searching for reliable biomarkers in this field is still needed^2,4^. Recently, advanced neuro-imaging techniques allowing for a deeper insight into the pathology of MS^15^ have attracted more attention in the context of prediction of disease activity or disability progression during immunomodulatory treatment^12,14,16^.

Response to treatment has traditionally been evaluated based on clinical (relapse rate, disability progression) and radiological (presence of demyelinating plaques in MRI—magnetic resonance imaging) aspects^17^. These were quantified using composite scores, including i.a. Rio score^18^, modified Rio score^19^ and MAGNIMS score^20^. Recently, the concept of NEDA (no evidence of disease activity) has emerged as a possible treatment goal^2^ used in clinical trials^21,22^, but also in real-world assessment^23^. NEDA, introduced in 2009^22^, was defined as a combination of three related measures of disease activity: (a) no relapses; (b) no disability progression and (c) no MRI activity (new or enlarging T2 lesions or gadolinium-enhancing lesions)^24^.

Diffusion-weighted imaging (DWI) is a nonconventional imaging method based on random movements of water molecules in extracellular space^15^. Diffusion may be measured using i.a. a mathematically calculated apparent diffusion coefficient (ADC), which detects changes in the overall diffusion irrespective of the motion direction^25,26^. Even subtle impairment of tissue architecture may result in facilitated diffusion, which is detectable as increased ADC or mean diffusivity (MD) values, as well as decreased fractional anisotropy (FA) values^15,16,27^. It was reported in a radiologic-histopathologic study that facilitated diffusion in normal-appearing white and grey matter (NAWM, NAGM) beyond demyelinating plaques in MS patients is related to neurodegeneration, and such changes are missed on conventional MR images^28^.

We designed a study aimed at investigating ADC measurements in selected regions of normal-appearing white and deep grey matter obtained during initial MRI with DWI in patients with relapsing–remitting MS as potential prognostic factors with regard to disease activity (NEDA status) during therapy with interferon-β in the 3-year follow-up, compared with conventional MRI data, demographic and clinical factors. Due to the lack of a control group of non-treated patients, we use the term “prognostic factors/indicators” of treatment response instead of “predictors”, according to Sormani^29^.

## Methods

### Study design

This was a retrospective, single-centre, independent, observational study approved by the Ethics Committee of Wroclaw Medical University, Poland (decision number 766/2017) and was conducted in accordance with the Helsinki Declaration. Each patient gave their informed and signed consent before participation in the MR examination.

### Group characteristics

There were 236 patients with MS studied in the Department of Radiology between 2008 and 2017.

Finally, the study comprised 87 patients (28 men, 59 women, aged 33.5 years at the beginning of treatment) with MS, who fulfilled the following inclusion criteria:

1. Patients with clinically defined relapsing–remitting course of MS, according to the 2010 revision of McDonald’s criteria^30^;
2. IFN-β as the only immunomodulatory treatment applied;
3. Availability of brain MRI examinations with appropriate DWI sequence performed before or after 1 year of treatment with IFN-β in the tertiary referral centre;
4. Up to 45 cerebral hyperintense T2/FLAIR lesions visible on conventional MRI;
5. No visible changes on conventional MRI sequences within the white and grey matter within regions of interest for ADC measurements.

**Figure 1:**
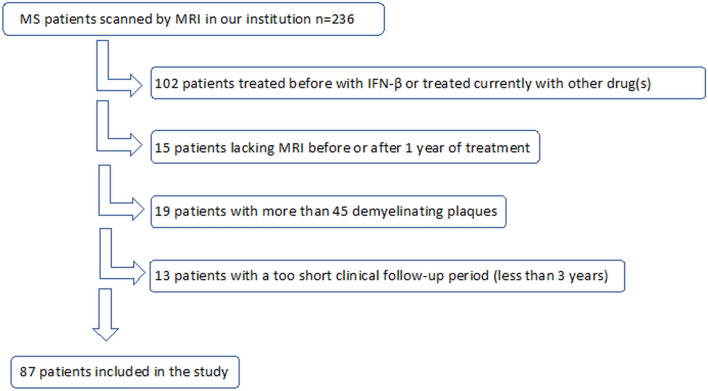
Study flow-chart.

All MS patients who did not fulfil the criteria mentioned above were excluded, which is depicted in the flow-chart (Fig. 1).

The patients were divided into two groups according to the timing of the initial MRI:

- MS0 group (n = 45)—1st MRI before starting treatment = true baseline MRI;
- MS1 group (n = 42)—1st MRI after 1 year of treatment with IFN-β and no available previous MRI scans = on-treatment MRI as baseline.

Demographic, clinical and conventional MRI characteristics of the sample are shown in detail in Table 1.

**Table 1:** Demographic, clinical and radiological data for relapsing–remitting MS patients treated with IFN-β in a 3-year follow-up.

Demographic, clinical and radiological characteristics	MS0 group—the 1st MRI before treatment with IFN-β—true baseline	MS1 group—the 1st MRI after 1 year of treatment with IFN-β—on-treatment MRI as baseline
The total number of analysed patients	45	42
Female:male (% of female)	33:12 (73.3%)	26:16 (61.9%)
Ratio	2.75:1	1.63:1
**Patients treated with [n of patients (%)]**		
Avonex	9 (20%)	8 (19%)
Betaferon	25 (55.6%)	27 (64.3%)
Rebif	11 (24.4%)	7 (16.7%)
Mean age at the onset of the disease in years ± SD (range)	32.5 ± 11.3 (18–58)	28.0 ± 7.9 (13–52)
Mean age at the 1st MRI in years ± SD (range)	35.0 ± 11.7 (19–61)	32.7 ± 7.8 (19–56)
Mean age at the beginning of treatment in years ± SD (range)	35.2 ± 11.6 (19–61)	31.7 ± 7.8 (18–55)
Mean time of the disease duration from the onset to treatment beginning in months ± SD (range)	35.7 ± 38.9 (2–156)	48.9 ± 42.5 (2–192)
Mean time of the disease duration from the onset to the 1st MRI in months ± SD (range)	32.1 ± 38.4 (1–144)	60.9 ± 42.5 (14–204)
Mean baseline EDSS ± SD	2.3 ± 1.3	2.0 ± 0.8
Median baseline EDSS (range)	2.0 (1–7)	2.0 (1–5)
Mean T2/FLAIR lesion number at the 1st MRI ± SD (range)	20.5 ± 12.9 (1–45)	19.3 ± 10.4 (3–45)
Patients with the 1st MRI after Gd-contrast administration [n, (%)]	43 (95.6%)	36 (85.7%)
Presence of Gd-enhancing lesions at the 1st MRI—[n of patients (% of patients with contrast administration)]	9 (20.9%)	3 (8.3%)
Mean number of Gd-enhancing lesions at the 1st MRI ± SD (range)	0.7 ± 2.0 (1–11)	0.1 ± 0.3 (1–1)
Presence of cervical spinal cord lesion at the 1st MRI—[n of patients (%)]	12 (26.7%)	5 (11.9%)
Mean time to loss of NEDA form the treatment beginning in the 3-years FU in month ± SD (range)	16.2 ± 8.4 (3–35)	NA
Mean time to loss of NEDA from the 1st MR in the 2-years FU in months ± SD (range)	NA	13.7 ± 4.4 (8–24)
Mean time to new lesion(s) formation from the treatment beginning in the 3-year FU (loss of “MRI” NEDA) in months ± SD (range)	18.5 ± 7.8 (8–36)	NA
Mean time to new lesion(s) formation from the 1st MRI in the 2-years FU (loss of “MRI” NEDA) in months ± SD (range)	NA	13.7 ± 3.8 (11–23)
Mean time to relapse from the treatment beginning in the 3-years FU (loss of “clinical” NEDA) in months ± SD (range)	13.7 ± 7.8 (3–25)	NA
Mean time to relapse from the 1st MRI in the 2-years FU (loss of “clinical” NEDA) in months ± SD (range)	NA	14.7 ± 6.2 (8–24)
Patients, who discontinued IFN-β treatment in the 3-year FU [n (%)]	9 (20%)	1 (2.4%)
Mean time of IFN-β treatment in the 3-years FU ± SD (range)	33.6 ± 5.4 (12–36)	35.8 ± 1.4 (27–36)
Patients, who switched treatment option in the 3-year FU [n (%)]	8 (17.8%)	0 (0%)
Mean time to alternative therapy in months ± SD (range)	23.5 ± 6.2 (12–30)	NA
**Reasons for loss of NEDA during treatment with IFN-β in the 3-year FU with [n of patients]**		
MRI activity	13	10
Clinical activity	10	8
Clinical and MRI activity	9	2

*MS* multiple sclerosis, *MRI* magnetic resonance imaging, *IFN-β* interferon-β, *SD* standard deviation, *EDSS* expanded disability status scale, *T2* T2-weigted images, MRI sequence, *FLAIR *fluid attenuation inversion recovery, MRI sequence, *NEDA* no evidence of disease activity, *Gd* gadolinium, *FU* follow-up, *N/A* not applicable.

Patients were treated with 3 available types of IFN-β—IFN-β-1a i.m. (Avonex), IFN-β-1b s.c. (Βetaferon) and IFN-β-1a s.c. (Rebif). Medication was continued within the 1st year of follow-up without change of treatment in all patients. Between the 1st and 3rd year of follow-up treatment was switched due to clinical and/or MRI disease activity in 17.8% (n = 8) of MS0 patients [to fingolimod (n = 3), glatiramer acetate (n = 3) and dimethyl fumarate (n = 2)]. In the MS1group none of the patients had their treatment switched during follow-up.

### NEDA definition and study outcomes

The main endpoints of our study was a NEDA failure, which was assessed after each year of IFN-β treatment in the MS0 group and within 1st–2nd and 1st–3rd year intervals for the MS1 group. In our study NEDA was defined as having no clinical relapses and/or no disability progression (“clinical” NEDA) and no new/enlarging or no gadolinium enhancing lesion(s) (“MRI” NEDA)^24^. A relapse was denoted as a monophasic (sub)acute clinical episode with symptoms and signs of neurological deficit, with a duration of at least 24 h, with or without recovery, and in the absence of fever or infection^31^. Since progression of the disability was observed only temporarily during relapses and in none of the patients sustained disability beyond relapses was noted, these clinical aspects of NEDA were not rated separately^17,21^. New and enlarged lesion(s) were assessed on T2/FLAIR and T1 post-contrast sequence^32^. The beginning of treatment with IFN-β was a start point for evaluation NEDA in the MS0 group with 1st MRI before therapy. In the MS1 group with 1st MRI after 1 year of treatment none of the patients had a relapse in the 1st year of treatment but history of new lesions formation was unknown in this period, which precluded us from using NEDA from the beginning of treatment—consequently in this group the start point for NEDA assessment was set at the 1st MRI. Three-years follow-up means that patients in both groups were clinically observed from the beginning of IFN-β treatment until 3 years had passed. However, because of different time points for starting NEDA assessment, the total length of MRI follow-up was at least 3 years for the MS0 group and 2 years for the MS1 group (from the 1st MRI after 1 year of treatment to finishing the 3-year follow-up procedure). Majority of the patients were assessed clinically and radiologically at least every 12 months from the onset of treatment until finishing the 3-year follow-up, unless a few of them omitted the last MRI control scan.

Endpoints for the MS0 group with 1st MRI before treatment are provided first and analogous endpoints for the MS1 group are depicted in round brackets, with subdivisions according to the statistical model used:

Model 1:

- loss of NEDA within the 1st year of treatment (not applicable for the MS1 group),
- loss of NEDA within the 2nd year of treatment (between the 1st and 2nd years of treatment),
- loss of NEDA within the 3rd year of treatment (between the 1st and 3rd years of treatment),
- loss of “MRI” NEDA within the 1st year of treatment (not applicable for the MS1 group),
- loss of “MRI” NEDA within the 2nd year of treatment (between the 1st and 2nd years of treatment),
- loss of “MRI” NEDA within the 3rd year of treatment (between the 1st and 3rd years of treatment),
- loss of “clinical” NEDA within the 1st year of treatment (not applicable for the MS1 group),
- loss of “clinical” NEDA within the 2nd year of treatment (between the 1st and 2nd years of treatment),
- loss of “clinical” NEDA within the 3rd year of treatment (between the 1st and 3rd years of treatment),
- treatment of IFN-β shorter than 3 years (the same),
- alternative treatment within 3 years of follow-up (not applicable for the MS1 group);

Model 2:

- time to loss of NEDA within 3 years of treatment (from the 1st MRI to 3 years of treatment),
- time to loss of “MRI” NEDA within 3 years of treatment (from the 1st MRI to 3 years of treatment),
- time to loss of “clinical” NEDA within 3 years of treatment (from the 1st MRI to 3 years of treatment),
- time to IFN-β treatment shorter than 3 years (the same),
- time to alternative treatment in 3 years of follow-up (not applicable for the MS1 group);

Model 3:

- reasons for loss of NEDA (the same).

### Potential prognostic factors of loss of NEDA and other related endpoints during IFN-β treatment

**Figure 2:**
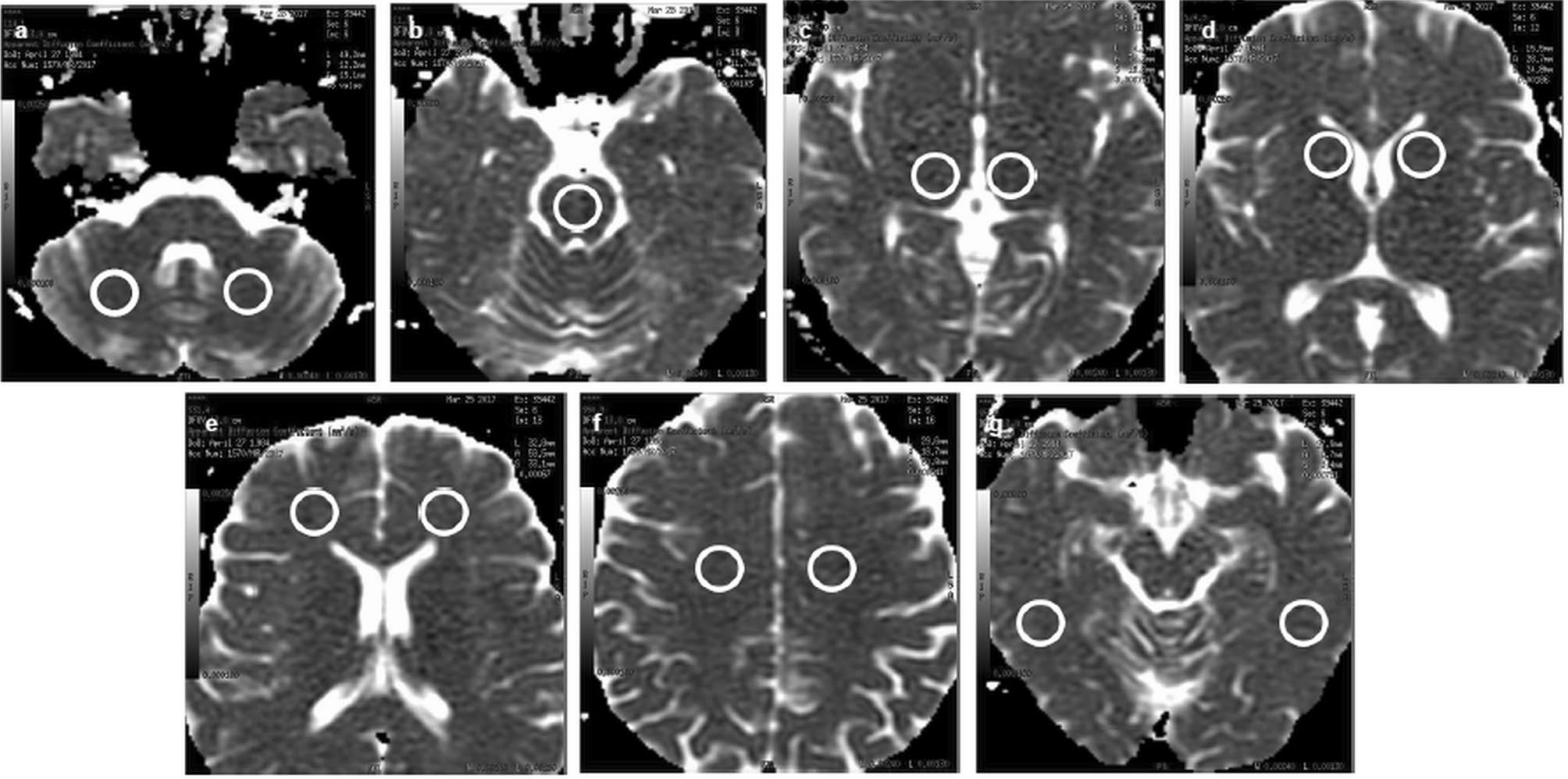
Representative ADC maps (transverse cross-section) indicating regions of interest (ROIs) placement. Measurements of ADC were obtained from the following regions of NAWM and NAGM: 1, 2: cerebellar white matter right and left, respectively (**a**). 3: pons (**b**). 4, 5: thalamus right and left, respectively (**c**). 6, 7: head of the caudate nuclei right and left, respectively (**d**). 8, 9: frontal white matter regions right and left, respectively (**e**). 10, 11: frontoparietal white matter at upper slices near the convexity right and left, respectively (**f**). 12, 13: temporal white matter of inferior temporal gyri right and left, respectively (**g**).

Demographic and clinical factors evaluated as potential prognostic indicators of all aforementioned endpoints during IFN-β treatment were as follows: sex, age at onset of the disease, age at the beginning of treatment, age at the 1st MRI, disease duration until the 1st MRI and onset of treatment and baseline EDSS. Investigated potential conventional and advanced radiological prognostic factors included:

- number of T2/FLAIR lesions at the 1st MRI—a total number of demyelinating plaques assessed on T2-weighted and FLAIR images,
- the presence and number of Gd-enhancing lesions on contrast-enhanced T1-weighted images at the 1st MRI,
- presence of demyelinating lesions in the cervical spine, evaluated on sagittal T2-weighted and contrast-enhanced T1-weighted images at the 1st MRI,
- value of ADC within the thirteen selected areas of NAWM and NAGM (Fig. 2).

In each MS patient the onset and disease duration were determined on the basis of medical records. Baseline disability was evaluated using the EDSS score, by an experienced neurologist.

Radiological parameters were obtained from the 1st MR examinations in MS patients before (MS0 group) or after the first year of treatment (MS1 group) and were assessed by an experienced radiologist, unaware of the clinical data, particularly the presence of future relapses or new lesions in future MR scans.

### Image acquisition

MR examinations were performed with a 1.5 T MR scanner (SignaHdx, GE Healthcare) using a 16-channel HNS coil. The conventional MR imaging protocol for MS patients consisted of axial T1-weighted SE, axial T2-weighted FSE, axial FLAIR, sagittal and coronal T2-weighted FRFSE, axial DWI SE/EPI sequence and post-gadolinium T1-weighted 3D-FSPGR images. DWI was performed using an axial single shot, spin-echo type echo-planar images with the following parameters: b = 0 and b = 1,000 mm^2^/s (TE 89.9 ms, TR 8,000 ms, FOV 26 cm, matrix 128 × 128), thickness: 5 mm, spacing: 0, time of acquisition: 40 s. We used the same method of image acquisition as described in our previously published paper^33^.

### Image analysis and post-processing

Diffusion-weighted imaging data were transferred to a GE Advantage Workstation 4.6. Functool software, provided by the manufacturer, was used in post-processing of images. ADC values were acquired by manually placing regions of interest (ROI), sized approx. 200 mm^2^, using DFOV = 13.0 cm on ADC maps in the following selected normal-appearing white and grey matter regions:

- 1, 2—right and left cerebellar white matter, respectively (Fig. 2a),
- 3—pons (Fig. 2b),
- 4, 5—right and left thalamus, respectively (Fig. 2c),
- 6, 7—right and left head of the caudate nuclei, respectively (Fig. 2d),
- 8, 9—right and left frontal white matter regions, respectively (Fig. 2e),
- 10, 11—right and left frontoparietal white matter at the upper slices near convexity, respectively (Fig. 2f),
- 12, 13—right and left temporal white matter of inferior temporal gyri, respectively (Fig. 2g).

T2-weighted images or FLAIR images were utilised to ensure that regions of interest did not include any demyelinating plaques or to exclude partial volume effect from border areas. We decided to choose many regions of interest in an attempt to obtain a more comprehensive insight in a potentially diffused pathology involving NAWM and NAGM. We used the same methods in image analysis and post-processing as we presented in our previously published study^33^.

### Statistical analysis

For binary outcomes (model 1), logistic regression was applied. Receiver operating characteristic (ROC) curves were used to choose the most appropriate cut-off value for a test of a binary classifier. In further analysis, the accuracy of the test was measured by the area under the curve (AUC)^34^. Sensitivity, specificity, predictive positive value (PPV) and negative predictive value (NPV) of the potential prognostic factors of NEDA loss and its components were calculated (model 1). In model 2 (time to reaching endpoint) Cox regression was used. In model 3 (prediction of reasons for NEDA loss) multinomial logistic regression was applied. The statistical outcomes were expressed by a classical odds ratio (OR), and hazard ratio (HR) together with confidence 95% intervals and p-values.

The false discovery rate correction (Benjamini-Hochberg) was added to adjust p-value.

Besides, cross-validation was performed using the Somers' Dxy rank discrimination index.

The computation was conducted in the R statistical platform^35^. P values < 0.05 were considered as statistically significant.

## Results

### Baseline demographic, clinical and MRI characteristics

Out of a total of 236 patients with a relapsing–remitting course of MS, 87 patients with complete clinical data at baseline and in the 3-year follow-up were included (Fig. 1), with further subdivision into a group of 45 patients with the initial MRI before treatment (MS0 group) and those with the 1st MRI after 1 year of treatment (n = 42, MS1 group). The whole baseline characteristics of both groups are summarised in Table 1. Two patients in the MS0 group and 6 patients in the MS1 group refused contrast administration at the 1st MRI, therefore they were not included in the assessment of presence and number of Gd-enhancing lesions at baseline. The sustaining of NEDA status was analysed until year 3 of observation if sufficient follow-up data was accessible. In the MS0 group, 2 patients were lost-to-follow in the 2nd year of observation, because of switching therapy due to first line treatment failure, and 9 patients were excluded from analysis in the 3rd year of follow-up due to treatment change or discontinuation. In the MS1 group, 3 patients were excluded for NEDA assessment between the 1st and 3rd year of treatment because of a lack of the MRI scan after 3 years of treatment and/or resignation of therapy (Table 2).

**Table 2:** Clinical, radiological and NEDA data of an appropriate period of time in MS patients, according to division into two groups, depending on the time of the 1st MRI as baseline scan.

	Within the 1st year of treatment	Within the 2nd year of treatment	Within the 3rd year of treatment
**MS0 group—the 1st MRI before treatment with IFN-β—baseline MRI**			
Analysed group of patients [n; (% of total baseline)]	45 (100%)	43 (95.6%)	36 (80%)
Patients lost-to-follow-up [n; (% of analysed)]	0 (0%)	2 (4.4%)	9 (20%)
NEDA maintenance [n; (% of analysed)]	29 (64.4%)	20 (46.5%)	12 (33.3%)
Loss of NEDA [n; (% of analysed)]	16 (35.6%)	23 (53.5%)	24 (67.7%)
Loss of “MRI” NEDA [n; (% of analysed)]	11 (24.4%)	18 (41.9%)	19 (52.8%)
Loss of “clinical” NEDA [n; (% of analysed)]	9 (19.6%)	12 (27.9%)	10 (27.8%)
Loss of NEDA due to one component [n; (% of analysed)]	12 (26.7%)	16 (37.2%)	19 (52.8%)
Loss of NEDA due to two components [n; (% of analysed)]	4 (8.9%)	7 (16.3%)	5 (13.9%)

	Within the 1st year of treatment	Between the 1st and 2nd years of treatment	Between the 1st and 3rd years of treatment
**MS1 group—the 1st MRI after 1 year of treatment with IFN-β only—on-treatment MRI as baseline**			
Analysed group of patients [n; (% of total baseline)]	N/A	42 (100%)	39 (92.9%)
Patients lost-to-follow-up [n; (% of analysed)]	N/A	0 (0%)	3 (7.7%)
NEDA maintenance [n; (% of analysed)]	N/A	30 (71.4%)	19 (48.7%)
Loss of NEDA [n; (% of analysed)]	N/A	12 (28.6%)	20 (51.3%)
Loss of “MRI” NEDA [n; (% of analysed)]	N/A	9 (21.4%)	14 (35.9%)
Loss of “clinical” NEDA [n; (% of analysed)]	N/A	5 (11.9%)	11 (28.2%)
Loss of NEDA due to one component [n; (% of analysed)]	N/A	10 (23.8%)	15 (38.5%)
Loss of NEDA due to two components [n; (% of analysed)]	N/A	2 (4.8%)	5 (12.8)

*MS* multiple sclerosis, *NEDA* no evidence of disease activity, *n* number, *IFN-β* interferon-β, *MRI* magnetic resonance imaging, *N/A* not applicable.

### NEDA at 1-, 2- and 3-year follow-up

In the MS0 group with baseline MRI before treatment at the 1st year of follow-up, 29 of 45 patients (64.4%) sustained NEDA and 16 patients (35.6%) lost NEDA. In detail, 11 patients (24.4%) lost NEDA due to MRI activity as a first finding and 9 patients failed due to clinical activity first. At the two-year follow-up, 43 patients were amenable to assessment, 20 (46.5%) maintained NEDA status, whereas 23 patients (53.5%) lost NEDA, in whom 18 patients (41.9%) did not sustain “MRI” NEDA first and 12 patients (27.9%) lost “clinical’ NEDA first. At the end of the 3-year follow-up period, 36 out of the initial 45 patients had appropriate data for analysis. Here, only 12 (33.3%) retained NEDA status, 19 patients (52.8%) presented MRI activity first and 10 patients (27.8%) developed relapses first. In the MS1 group with the 1st MRI after 1 year of treatment NEDA was assessed for periods between the 1st and 2nd and between the 1st and 3rd year of IFN-β treatment, because of a lack of baseline MRI obtained before treatment. Between the 1st and 2nd years of follow-up, 30 patients from 42 (71.4%) maintained NEDA status, 9 patients (21.4%) failed in “MRI” NEDA and 5 patients (11.9%) lost “clinical” NEDA at first. In the period between the 1st and 3rd years of treatment, 39 patients had sufficient clinical and radiological data for evaluation—19 patients (48.7%) sustained NEDA status, while 20 patients (51.3%) lost NEDA, including 14 patients (35.9%) with “MRI” NEDA loss, 11 patients (28.2%) with “clinical” NEDA loss as a first finding. In both groups and in any period of observation, a majority of patients lost NEDA due to failure in one component, which was mostly associated with formation of new lesions (Table 2).

### Best individual prognostic markers of loss of NEDA during treatment with IFN-β

We performed an exploratory multivariate analysis of 24 potentially valuable independent predictors of disease activity according to the NEDA concept in a 1-, 2- and 3-year follow-up.

#### Model 1—prognostic markers of loss of NEDA and its components

In the MS0 group with baseline MRI before treatment we found only one statistically significant prognostic marker of NEDA loss until the 3rd year of treatment—the ADC value in NAWM of the left cerebellar hemisphere (ROI 2), OR 1.03, p = 0.038. New lesions formation until the 1st year of treatment was predicted by only one factor as well—the ADC value in pons (ROI 3) with OR 1.02, p = 0.026. The most powerful factor in prognosis of loss of “MRI” NEDA was the presence of Gd-enhancing lesion(s) at baseline scan—patients with this feature were almost at a ninefold higher risk of loss of “MRI” NEDA during 2 years of IFN-β treatment.

In the MS1 group with baseline MRI after 1 year of INF-β treatment we found that an increased ADC value in the 1st MRI in the left frontal NAWM (ROI 9), the right and left frontoparietal NAWM (ROI 10 and 11) and in the right temporal NAWM (ROI 12) predicted loss of NEDA between the 1st and 3rd years of treatment with OR 1.02 (p = 0.017–0.047). ADC values obtained from the right and left frontoparietal NAWM (ROI 10 and 11) and from the left temporal NAWM (ROI 13) were significant factors in prognosis of “clinical” NEDA loss between the 1st and 2nd year of treatment (OR 1.02–1.03, p = 0.0350–0.048), reaching the highest validation index (Dxy 0.61–0.63) among all investigated prognostic factors. Baseline number of T2/FLAIR lesions was associated with the slightly higher risk (OR 1.11, p = 0.008) than the increased ADC value in the right and left frontal NAWM (ROI 8 and 9) and in the right and left frontoparietal NAWM (ROI 10 and 11) (OR 1.02–1.03, p = 0.004–0.01) in the prognosis of “clinical” NEDA failure between the 1st and 3rd year of treatment. These factors achieved moderately high validation performance as well (Dxy 0.54–0.63). Other evaluated potential factors showed no statistical significance as prognostic markers of disease activity during 1st line treatment in the 3-year follow-up. None of the factors predicted treatment with IFN-β shorter than 3 years or treatment change in both groups. (Table 3, Suppl. Fig. S1).

**Table 3:** Logistic regression analysis of the association between significant MRI prognostic markers and endpoints related to disease activity (model 1).

Type of endpoint	Prognostic marker	OR	CI 95%	Raw p-value	Adjusted p-value	Somers’ Dxy rank discrimination index
**MS0 group—the 1st MRI before treatment with IFN-β—baseline MRI**						
Loss of NEDA within the 1st year of treatment	None	–	–	–	–	–
Loss of NEDA within the 2nd year of treatment	None	–	–	–	–	–
Loss of NEDA within the 3rd year of treatment	ADC in ROI 2—left cerebellar white matter	1.03	1.00, 1.06	0.038	0.042	0.47
Loss of “MRI” within the 1st year of treatment	ADC in ROI 3—pons	1.02	1.00, 1.05	0.026	0.034	0.39
Loss of “MRI” NEDA within the 2nd year of treatment	Presence of Gd-enhancing lesion at 1st MRI	8.94	1.55, 51.48	0.014	0.024	0.36
Loss of “MRI” NEDA within the 3rd year of treatment	None	–	–	–	–	–
Loss of “clinical” NEDA within the 1st year of treatment	None	–	–	–	–	–
Loss of “clinical” NEDA within the 2nd year of treatment	None	–	–	–	–	–
Loss of “clinical” NEDA within the 3rd year of treatment	None	–	–	–	–	–
Treatment with IFN-β shorter than 3 years	None	–	–	–	–	–
Alternative treatment before 3 years of treatment	None	–	–	–	–	–
**MS1 group—the 1st MRI after 1 year of treatment with IFN-β only—on-treatment MRI as baseline**						
Loss of NEDA between the 1st and 2nd years of treatment	None	–	–	–	–	–
Loss of NEDA between the 1st and 3rd years of treatment	ADC in ROI 9—left frontal white matter	1.02	1.00, 1.04	0.017	0.026	0.47
	ADC in ROI 10—right frontoparietal white matter	1.02	1.00, 1.03	0.028	0.035	0.44
	ADC in ROI 11—left frontoparietal white matter	1.02	1.00, 1.03	0.047	0.048	0.37
	ADC in ROI 12—right temporal white matter	1.02	1.00, 1.04	0.024	0.032	0.44
Loss of “MRI” NEDA between the 1st and 2nd years of treatment	None	–	–	–	–	–
Loss of “MRI” NEDA between the 1st and 3rd years of treatment	None	–	–	–	–	–
Loss of “clinical” NEDA between the 1st and 2nd years of treatment	ADC in ROI 10—right frontoparietal white matter	1.02	1.00, 1.04	0.042	0.045	**0.63**
	ADC in ROI 11—left frontoparietal white matter	1.02	1.00, 1.04	0.048	0.048	**0.61**
	ADC in ROI 13—left temporal white matter	1.03	1.00, 1.06	0.035	0.041	**0.62**
Loss of “clinical” NEDA between the 1st and 3rd years of treatment	Number of T2/FLAIR lesions at 1st MRI	1.11	1.03, 1.21	0.008	0.018	**0.55**
	ADC in ROI 8—right frontal white matter	1.03	1.01, 1.04	0.009	0.019	**0.54**
	ADC in ROI 9—left frontal white matter	1.03	1.01, 1.04	0.01	0.019	**0.57**
	ADC in ROI 10—right frontoparietal white matter	1.03	1.01, 1.04	0.004	0.015	**0.63**
	ADC in ROI 11—left frontoparietal white matter	1.02	1.01, 1.04	0.005	0.015	**0.60**
Treatment with IFN-β shorter than 3 years	None	–	–	–	–	–
Alternative treatment before 3 years of treatment	None	–	–	–	–	–

Presence of the Gd-enhancing lesion at baseline MRI was the strongest predictor of “MRI” NEDA loss in the MS0 group. ADC measurements obtained from the 1st MRI after 1 year of treatment (MS1 group) were most the common significant markers in prognosis of NEDA loss and “clinical” NEDA loss in the subsequent 2 years of IFN-β treatment, with the best performance in a cross-validation procedure, reaching the Somers’ Dxy rank discrimination index > 0.6 for the latter endpoints (marked in bold). None of the other baseline demographic or clinical factors increased the risk of NEDA failure and its components in both groups.

*MS* multiple sclerosis, *OR* odds ratio, *CI* confidence interval, *IFN-β* interferon-β, *NEDA* no evidence of disease activity, *ADC* apparent diffusion coefficient, *ROI* region of interest, *ROI 8, 9* frontal white matter regions right and left, respectively, *ROI 10, 11* frontoparietal white matter right and left, respectively, *ROI 12, 13* right and left temporal white matter, respectively, *T2* T2-weighted images, MRI sequence, *FLAIR* fluid attenuation inversion recovery, MRI sequence.

Based on the ROC curves (Suppl. Fig. S2) analysis, accuracy for different cut-off values of the significant indicators of disease activity was evaluated, which is shown in detail in Table 4. The highest accuracy was achieved in prognosis of loss of “clinical” NEDA between the 1st and 2nd year of treatment in the MS1 group for ADC measurements in the right and left frontoparietal NAWM (ROI 10 and 11) and the left temporal NAWM (ROI 13) (AUC 80.3–81.6%, sensitivity 80–100%, specificity 64.9–75.5%) as well as in prognosis of loss of “clinical” NEDA between the 1st and 3rd year of treatment in the same group for the ADC value in the right frontoparietal NAWM (ROI 10) (AUC 81.4%, sensitivity 72.2%, specificity 83.9%). Other aforementioned significant prognostic factors of disease activity in both groups reached moderate accuracy as single measurements (68.4–79.8%). None of the combinations between significant factors gained accuracy over the single measure.

**Table 4:** Results of ROC analyses for significant prognostic markers of endpoints related to disease activity during IFN-β treatment in a 3 years follow-up and their statistically significant combinations.

Type of endpoint	Prognostic markers	Cut-off	AUC	SP	SN	NPV	PPV
**MS0 group—the 1st MRI before treatment with IFN-β—before-treatment MRI as baseline**							
Loss of NEDA within the 3rd year of treatment	ADC in ROI 2—left cerebellar white matter	748.0 × 10^–6^ mm^2^/s	73.3	**91.7**	54.2	50.0	**92.9**
Loss of “MRI” NEDA within the 1st year of treatment	ADC in ROI 3—pons	784.5 × 10^–6^ mm^2^/s	69.7	**94.0**	45.0	**84.2**	71.4
**MS1 group—the 1st MRI after 1 year of treatment with IFN-β only—on-treatment MRI as baseline**							
Loss of NEDA between the 1st and 3rd years of treatment	ADC in ROI 9—left frontal white matter	790 × 10^–6^ mm^2^/s	73.7	**94.7**	60.0	69.2	**92.3**
	ADC in ROI 10—right frontoparietal white matter	689 × 10^–6^ mm^2^/s	72.1	63.2	**80.0**	75.0	69.6
	ADC in ROI 11—left frontoparietal white matter	702.5 × 10^–6^ mm^2^/s	68.4	73.7	65.0	66.7	72.2
	ADC in ROI 12—right temporal white matter	771.5 × 10^–6^ mm^2^/s	72.0	63.2	**80.0**	75.0	69.6
	ADC in ROI 9 + 10	N/A	66.1	77.4	63.6	60.0	**80.0**
	ADC in ROI 9 + 11	N/A	67.6	74.2	65.9	60.5	78.4
	ADC in ROI 9 + 10 + 11	N/A	67.4	67.7	72.7	**90.4**	39.2
	ADC in ROI 9 + 12	N/A	67.8	77.4	63.6	60.0	**80.0**
	ADC in ROI 10 + 12	N/A	65.5	51.6	**81.8**	66.7	70.6
	ADC in ROI 11 + 12	N/A	65.2	67.7	63.6	56.8	73.7
	ADC in ROI 10 + 11 + 12	N/A	65.7	51.6	**81.8**	66.7	70.6
	ADC in ROI 9 + 10 + 12	N/A	67.8	74.2	63.6	59.0	77.8
	ADC in ROI 9 + 11 + 12	N/A	68.1	71.0	66.0	59.5	76.3
	ADC in ROI 9 + 10 + 11 + 12	N/A	68.3	67.7	72.7	63.6	76.2
Loss of “clinical” NEDA between the 1st and 2nd years of treatment	ADC in ROI 10—right frontoparietal white matter	721.5 × 10^–6^ mm^2^/s	**81.6**	75.7	**80.0**	**96.6**	30.8
	ADC in ROI 11—left frontoparietal white matter	709.5 × 10^–6^ mm^2^/s	**80.3**	64.9	**100.0**	**100.0**	27.8
	ADC in ROI 13—left temporal white matter	785.5 × 10^–6^ mm^2^/s	**81.1**	64.9	**100.0**	**100.0**	27.8
Loss of “clinical” NEDA between 1st and 3rd years of treatment	number of T2/FLAIR lesions at 1st MRI	25.5	77.3	**90.3**	63.6	**87.5**	70.0
	ADC in ROI 8—right frontal white matter	779 × 10^–6^ mm^2^/s	77.0	77.4	72.7	**88.9**	53.3
	ADC in ROI 9—left frontal white matter	790 × 10^–6^ mm^2^/s	78.5	**83.9**	**81.8**	**92.9**	64.3
	ADC in ROI 10—right frontoparietal white matter	721.5 × 10^–6^ mm^2^/s	**81.4**	**83.9**	72.7	**89.7**	61.5
	ADC in ROI 11—left frontoparietal white matter	702.5 × 10^–6^ mm^2^/s	79.8	67.7	**90.9**	**95.5**	50.0
	ADC in ROI 8 + 9	N/A	74.9	77.2	66.7	**86.3**	51.9
	ADC in ROI 10 + 11	N/A	67.4	57.9	76.2	**86.8**	40.0
	ADC in ROI 8 + 10	N/A	73.6	73.7	71.4	**87.5**	50.0
	ADC in ROI 8 + 11	N/A	73.9	73.7	71.4	**87.5**	50.0
	ADC in ROI 9 + 10	N/A	69.8	64.9	**81.0**	**90.2**	45.9
	ADC in ROI 9 + 11	N/A	69.8	64.9	**81.0**	**90.2**	45.9
	ADC in ROI 8 + 9 + 10	N/A	75.3	75.4	71.4	**87.8**	51.7
	ADC in ROI 8 + 9 + 11	N/A	75.7	**89.5**	42.9	**81.0**	61.2
	ADC in ROI 8 + 10 + 11	N/A	74.3	78.9	66.7	**86.5**	53.8
	ADC in ROI 9 + 10 + 11	N/A	70.3	66.7	81.0	**90.5**	47.2
	ADC in ROI 8 + 9 + 10 + 11	N/A	75.6	**87.7**	57.1	**84.7**	63.2

Single markers achieved higher AUC than their combinations. The best parameters attaining ≥ 80% are marked in bold.

*ROC* receiver operating characteristic, *MS* multiple sclerosis, *IFN-β* interferon-β, *AUC* area under the curve, accuracy, *SP* specificity, *SN* sensitivity, *NPV* negative predictive value, *PPV* positive predictive value, *NEDA* no evidence of disease activity, *ADC* apparent diffusion coefficient, *ROI* region of interest, *N/A* not applicable, *ROI 8, 9* frontal white matter regions right and left, respectively, *ROI 10, 11* frontoparietal white matter right and left, respectively, *ROI 12, 13* right and left temporal white matter, respectively, *T2* T2-weighted images, MRI sequence, *FLAIR* fluid attenuation inversion recovery, MRI sequence.

#### Model 2—prognosis of time to NEDA loss and its components

MS0 patients with baseline presence of the Gd-enhancing lesion had a 2.9-fold risk of shorter time to reach loss of NEDA (p = 0.0097) and a 4.8-fold risk to reach loss of “MRI” NEDA (p = 0.0004) in the 3-year follow-up. Time to loss of “MRI” NEDA was also connected with the baseline number of the Gd-enhancing lesion with a 1.25 hazard ratio (p = 0.0048) and an ADC value in pontine NAWM with a 1.01 hazard ratio (p = 0.038). Baseline EDSS increased the risk of reaching loss of “clinical” NEDA up to 1.32 times (p = 0.032). All mentioned factors were associated with a slight to low cross-validation index (Dxy = 0.17–0.31).

In the MS1 group, measurements of ADC in a few particular locations at the 1st MR after 1 year of treatment played a role in prediction of the time to reach loss of NEDA and together with the number of T2/FLAIR lesions at the 1st MRI in prediction of the time to loss of “clinical” NEDA. All of the ADC values obtained from both frontal NAWM regions (ROI 8 and 9), both frontoparietal NAWM regions (ROI 10 and 11) and from the right temporal NAWM region (ROI 12) were associated with 1.01 HR (p = 0.0056–0.045). The number of T2/FLAIR gave about a 6% higher risk of reaching loss of “clinical” NEDA. These factors were found to have low to moderate cross-validation performance. No other factors increased the risk of reaching the time point of NEDA failure and its components, as well as none of them predicted the time of IFN-β treatment shorter than 3 years or the time to alternative treatment in both groups (Table 5, Suppl. Fig. S3).

**Table 5:** Cox regression analysis of the associations between the most crucial demographic, clinical and MRI risk factors of time to loss of NEDA and its components (model 2).

Type of endpoint	Risk factor	HR	CI 95%	Raw p-value	Adjusted p-value	Somers’ Dxy rank discrimination index
**MS0 group—the 1st MRI before treatment with IFN-β–baseline MRI**						
Time to loss of NEDA from the treatment beginning to 3 years of treatment	Baseline presence of Gd + lesion(s)	2.9	1.29, 6.50	0.00967	0.02	0.1679
Time to loss of “MRI” NEDA from the treatment beginning to 3 years of treatment	Baseline presence of Gd + lesion(s)	4.77	2.00, 11.4	0.000432	0.002	0.2813
	Baseline number of Gd + lesion(s)	1.25	1.07, 1.46	0.00475	0.015	0.2755
	ADC in ROI 3—pons	1.01	1.001, 1.021	0.0383	0.042	0.1781
Time to loss of “clinical” NEDA from the treatment beginning to 3 years of treatment	Baseline EDSS	1.37	1.03, 1.82	0.0315	0.039	0.3092
Time of treatment with IFN-β shorter than 3 years	None	–	–	–		
Time to alternative treatment in the 3-years FU	None	–	–	–		
**MS1 group—the 1st MRI after 1 year of treatment with IFN-β only—on-treatment MRI as baseline**						
Time to loss of NEDA from the 1st MRI to 3 years of treatment	ADC in ROI 9—left frontal white matter	1.01	1.003, 1.022	0.0118	0.022	0.3399
	ADC in ROI 10—right frontoparietal white matter	1.01	1.004, 1.021	0.00561	0.015	0.3513
	ADC in ROI 11—left frontoparietal white matter	1.01	1.002, 1.019	0.0179	0.027	0.2598
	ADC in ROI 12—right temporal white matter	1.01	1.001, 1.027	0.0339	0.040	0.2696
Time to loss of “MRI” NEDA from the 1st MRI to 3 years of treatment	None	–	–	–		
Time to loss of “clinical” NEDA from the 1st MRI to 3 years of treatment	T2/FLAIR lesions number at 1st MRI	1.06	1.01, 1.12	0.0222	0.035	0.3802
	ADC in ROI 8—right frontal white matter	1.01	1.001, 1.026	0.0375	0.042	0.3802
	ADC in ROI 9—left frontal white matter	1.01	1.000, 1.026	0.0454	0.048	0.4353
	ADC in ROI 10—right frontoparietal white matter	1.01	1.003, 1.024	0.0148	0.025	0.4904
	ADC in ROI 11—left frontoparietal white matter	1.01	1.002, 1.022	0.022	0.03	0.4490
Time of treatment with IFN-β shorter than 3 years	None	–	–	–		
Time to alternative treatment in the 3-years FU	None	–	–	–		

In the MS0 group the presence of the Gd-enhancing lesion at baseline MRI increased almost 3-times the risk of reaching loss of NEDA and almost 5-times the risk of reaching loss of “MRI” NEDA. In the MS1 group ADC values in supratentorial areas obtained at MRI after 1 year of treatment increased the risk of loss of NEDA and its clinical component by about 1%

*MS* multiple sclerosis, *HR* hazard ratio, *EDSS* expanded disability status scale, *NEDA* no evidence of disease activity, *Gd* gadolinium, *Gd* + gadolinium-enhancing, *ADC* apparent diffusion coefficient, *ROI* region of interest, *ROI 3* pons, *ROI 8, 9* frontal white matter regions right and left, respectively, *ROI 10 11* frontoparietal white matter right and left, respectively, *ROI 12, 13* temporal white matter right and left, respectively, *T2* T2-weighted images, *MRI* sequence, *FLAIR* fluid attenuation inversion recovery, MRI sequence, *FU* follow-up.

#### Model 3—prognostic factors of the reasons for NEDA loss

The assessment of reasons for NEDA failure until the end of the 3-year follow-up for all 32 patients with loss of NEDA in the MS0 group showed that NEDA loss resulted from subclinical disease activity in MRI in only 13 patients (40.6% of all patients with NEDA loss), clinical activity in only 10 patients (31.3%), while in 9 patients (28.1%) NEDA failure was a consequence of loss of both components. In the MS1 group a total number of 20 patients experienced NEDA failure, for the following reasons—loss of “MRI” NEDA in 10 patients (50%), loss of “clinical” NEDA in 8 patients (40%) and a combination of both components in 2 patients (20%) (Table 1). None of the 24 analysed potential factors predicted the reason of loss of NEDA in both groups of MS patients.

### Benjamini–Hochberg correction

In analysis of the false discovery rate correction, all provided potential predictors remained statistically significant with the adjusted p value < 0.05—data are summarised in Tables 3 and 5.

### Cross-validation procedure

In the course of cross-validation procedure we found that the best performance with the Somers' Dxy rank discrimination index was achieved in model 1 in MS1 group in prediction of the “clinical” NEDA loss in both evaluated periods (1st–2nd and 1st–3rd year of IFN-β treatment), reaching moderately high index (Dxy = 0.54–0.63) (Table 3). Apart from that majority of previously described statistically significant prognostic factors were characterised by low to moderate index (Dxy = 0.31–0.49). Details are provided in the last column of Table 3 and 5.

## Discussion

The prompt identification of MS patients with insufficient response to disease-modifying treatment is pivotal for selecting candidates to change the therapy, aiming at an individualised treatment strategy for optimal control of the disease^2,4^. The fundamental challenge in the evaluation of potential predictors is the lack of a shared definition of treatment response in MS^2^. Non-response to treatment is usually assessed on the basis of one of three outcomes or their combination: disability progression^5,11,14,16^, occurrence of relapses^11,12^, and/or presence of new/active MRI lesions^19^. However, in the literature variable criteria based on these responses have been used within different follow-up procedures^4^. NEDA is a concept introduced in clinical trials of natalizumab in 2009 by Havrdova et al., as a surrogate for treatment response based on no evidence of disease activity neither clinical (relapses, worsening disability) nor subclinical (new MRI lesions)^22^. Nowadays, some modifications to the primary definitions have been made, with addition of the fourth and fifth domain—pathological volume loss of the brain and raised cerebrospinal fluid neurofilament level, respectively^24^. NEDA is increasingly used in real-world datasets^23^, nevertheless there are some flaws in using NEDA as a goal of treatment—the main drawback is that each NEDA component is equal. As a consequence, a new clinically silent lesion in MRI is as important as the occurrence of a severely disabling relapse^36^.

In our study we applied NEDA criteria retrospectively in the RRMS cohort treated with IFN-β as a first-line therapy. We demonstrated NEDA rates of 64.4%, 46.5% and 33.3% after the 1st, 2nd and 3rdyear of treatment, respectively in patients with the initial MRI before therapy, as well as 71.4% and 48.7% for periods 1st–2nd and 1st–3rd years of treatment, respectively, in patients with the 1st MRI after 1 year of treatment (Table 2). Higher rates of NEDA maintenance in the second group (MS1) may result from underestimation of the first year of therapy, when it was not amenable to assess the formation of new lesions, due to a lack of MRI data. Besides, we received higher rates of NEDA maintenance in both groups compared to the study of Huhn et al., where rates of 45%, 29%, and 21% were achieved at each analogical year of treatment^23^ compared with our MS0 group. Apart from a known individual variability in the course of MS, these differences may result from distinctive inclusion criteria—we analysed patients treated with IFN-β only, whereas in the cited study patients treated with various drugs were included^23^.

Commonly reported predictors or prognostic factors of treatment response include age^5^, sex, disease duration^6^, baseline disability^6,8^, number of relapses during the first year of treatment^9^, presence and/or number and/or volume of new lesions on T2-weighted images or gadolinium-enhancing lesions^8–14^. In our cohort we found a few relations between some of these factors and risk of NEDA loss. In the MS0 group the presence of Gd-enhancing lesion(s) at baseline scan was the best predictor of “MRI” NEDA loss until the 2nd year of therapy (OR 8.94), time to NEDA loss (HR 2.9) and time to “MRI” NEDA loss (HR 4.77). Apart from that, baseline EDSS predicted time to loss of “clinical” NEDA with a 1.37 hazard ratio. However, all of them reached slight to low validation index (Dxy 0.17–0.36). In the MS1 group, the number of T2/FLAIR lesions at the 1st MRI favoured loss of “clinical” NEDA between the 1st and 3rd years of therapy (OR 1.11, Dxy 0.55) and increased the risk of time to loss of “clinical” NEDA (HR 1.06, Dxy 0.38) (Tables 3 and 5).

As there is a scarcity of data regarding the use of DWI measurements in the prognosis of IFN-β treatment response^16^, we decided to evaluate the usefulness of easily accessible ADC measurements in this role. Involvement of so-called normal-appearing white and grey matter by a pathological process even in early diagnosed MS patients has been commonly investigated^16,27,37,38^ and a connection with that detected by advanced MRI techniques facilitated diffusion was made^28^. This observation is mainly explained by three phenomena in the literature: firstly, conventional MRI sequences used at 1.5 T or 3 T may be deficient in demonstrating all demyelinate lesions in both white and grey matter—the use of higher field strength devices enables the detection of more plaques in regions supposed to be normal on conventional MRI^37^. Secondly, diffusion derangements can predate the formation of new demyelinate plaques^38^. Thirdly, even though MS is regarded as a primarily demyelinating disease, the axonopathy occurs parallel beyond the plaques even at the early stage of the disease^38,39^, which may be related to Wallerian degeneration, in which axons traversing focal lesions are damaged even at a far distance from the demyelinating plaque^40,41^. Retrogenesis is the other hypothesis, assuming that late-myelinating fibres are more prone to age-related and disease-related degeneration and consequently, they are more likely to show diffusion alterations first^42^.

According to our analysis, ADC measurements in some regions are connected with a statistically significant higher risk of loss of NEDA and its components. In a group of patients with baseline MRI before treatment, we found that ADC measured in infratentorial white matter regions (ROIs 1–3, cerebellum and pons) predicted loss of NEDA, loss of “MRI” NEDA (model 1) and time to “MRI” NEDA loss (model 2), but with slight to moderate validation coefficient (Dxy 0.18–0.47). Conversely, in patients with the 1st MRI after 1 year of treatment, supratentorial ADC measurements (ROIs 8–13—frontal, frontoparietal and temporal NAWM) escalated the risk of NEDA loss between the 1st and 3rd year of treatment and “clinical” NEDA loss between the 1st and 2nd, reaching the highest accuracy ≥ 80% and the highest performance in cross-validation (Dxy 0.61–0.63) among investigated factors. ADC measurements in these locations predicted also the “clinical” NEDA failure between the 1st and 3rd year of treatment with a little lower accuracy of 77.0–81.4% and validation coefficient (Dxy 0.54–0.63). In model 2, time to loss of NEDA and its “clinical” component was predicted by ADC obtained from the supratentorial NAWM regions as well. Each ADC value in both groups was associated with an increase in appropriate risk (OR or HR) between 1–3% (Tables 3 and 5), which was far lower than the increase of some clinical and conventional MRI factors (e.g. the presence of a Gd-enhancing lesion), but, important to note, an increase in just one step of ADC value (e.g. from 779 to 780 × 10^–6^ mm^2^/s) implicated escalation of a risk in 1–3%. Apart from that most other investigated factors were not useable in predicting any of the seventeen proposed outcomes, whereas ADC measurements did.

There are some limitations of our study. Firstly, the study sample is small—we could include only 87 from all 236 patients managed at our institution because of the tight inclusion criteria. Secondly, we had only 45 patients with true baseline MRI before treatment, however we found that ADC measurements in on-treatment MRI are even more commonly and in better degree associated with statistically significant prediction of subsequent loss of NEDA, particularly the loss of “clinical” NEDA. Thirdly, ADC measurements cannot be applied to all MS patients—patients with numerous plaques and plaques in regions of interest are not amenable to this method. On the other hand, we provided some novelty by using ADC measurements in the prognosis of disease activity during IFN-β treatment as a simple and easy tool.

## Conclusions

In conclusion, our results seem to suggest that the ADC measurements of NAWM, particularly in the frontal, frontoparietal and temporal regions obtained after 1 year of treatment, may contribute to the prognosis of MS activity according to the NEDA concept during interferon-β therapy. This is a relatively easy imaging measure, that might be incorporated into standard follow-up procedures in MS patients. However, further research of this method should be performed before its widespread implementation.

## Supplementary information

Supplementary file 1.

## Data Availability

Data will be available on demand, there is no data related to this article in data repositories.
