# Supplementary material for: The role of ADC values within the normal-appearing brain in the prognosis of multiple sclerosis activity during interferon-β therapy in the 3-year follow-up: a preliminary report
Source: Sci Rep. 2020 Jul 30;10:12828. doi: 10.1038/s41598-020-69383-3 (PMC7393067; doi:10.1038/s41598-020-69383-3)
Supplement: Supplementary file 1 [file 41598_2020_69383_MOESM1_ESM.pdf]

Supplementary figures

Title: The role of ADC values within the normal appearing brain in the prognosis of multiple sclerosis activity during interferon-β therapy in the 3-year follow-up – a preliminary report

Authors: Anna Zacharzewska-Gondek, Anna Pokryszko-Dragan, Sławomir Budrewicz, Marek Sąsiadek, Grzegorz Trybek, Joanna Bladowska

Suppl. Fig. S1. Statistically significant results of logistic regression model analysis (model 1). There are three prognostic factors – ADC in the left cerebellar white matter, ADC in the pons and presence of the Gd-enhancing lesion predicting loss of NEDA until the 3rd year, loss of "MRI" NEDA until the 1st and until the 2nd year of treatment in the MS0 group with the 1st MRI before treatment, respectively (**a**). In the MS1 group with the 1st MRI after 1 year of therapy, loss of NEDA between the 1st and 3rd year of treatment was predicted by ADC values in the left frontal, the right and left frontoparietal and in the right temporal NAWM (**b**), loss of "clinical" NEDA between the 1st and 2nd year of treatment by the ADC value in the right and left frontoparietal and the left temporal NAWM (**c**) and loss of "clinical" NEDA between the 1st and 3rd year of treatment was predicted by the baseline number of T2/FLAIR lesions, the ADC value in the right and left frontal and in the right and left frontoparietal NAWM (**d**). Details are provided in Table 3.

a)

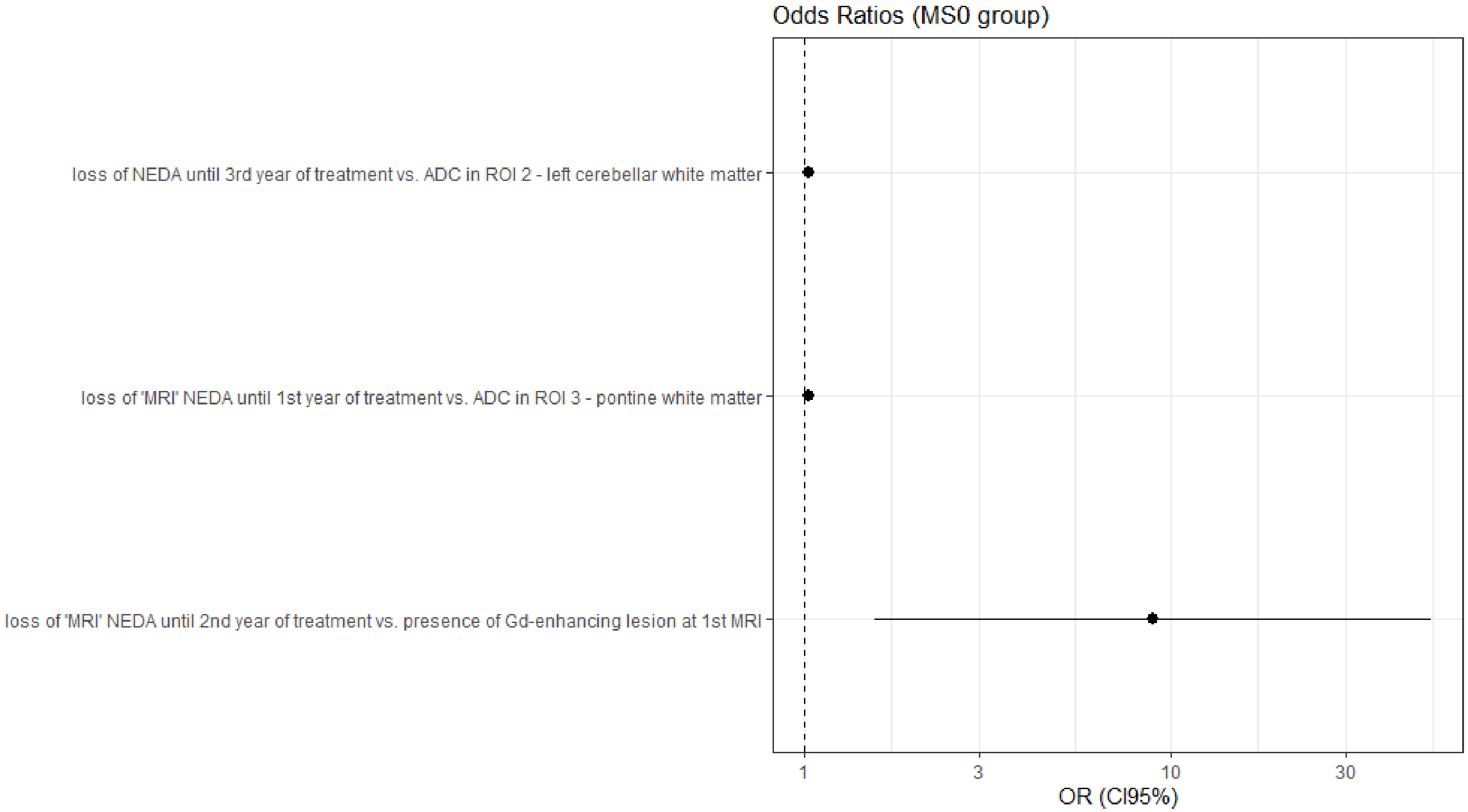

b)

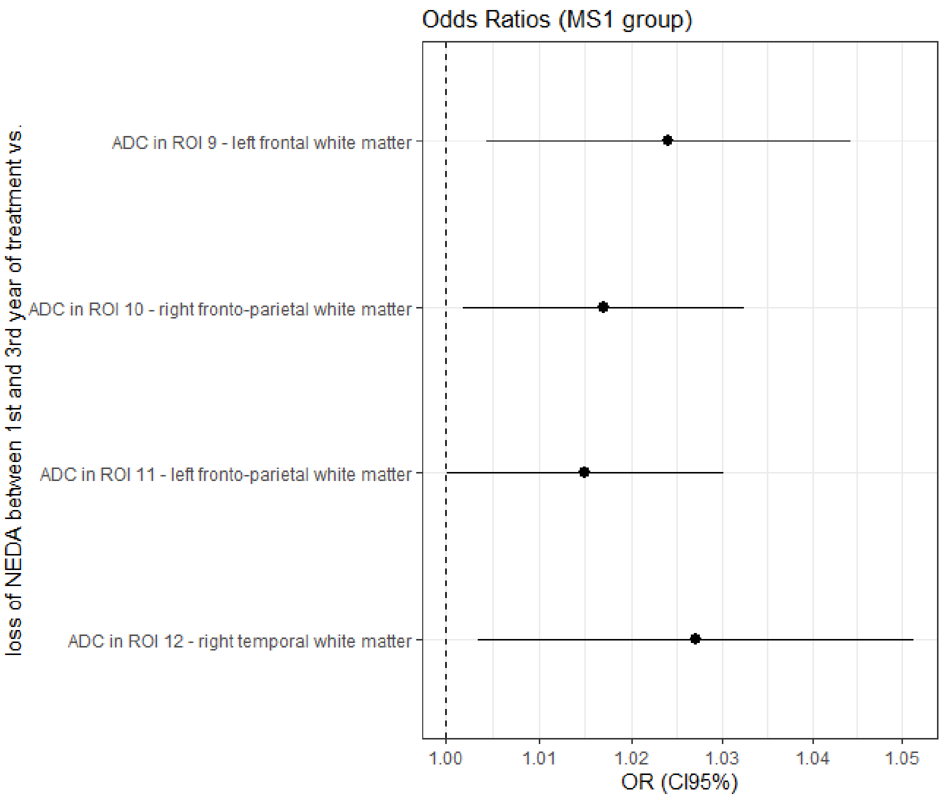
Odds Ratios (MS1 group)
loss of NEDA between 1st and 3rd year of treatment vs.
ADC in ROI 9 - left frontal white matter
ADC in ROI 10 - right fronto-parietal white matter
ADC in ROI 11 - left fronto-parietal white matter
ADC in ROI 12 - right temporal white matter
1.00
1.01
1.02
1.03
1.04
1.05
OR (CI95%)

c)

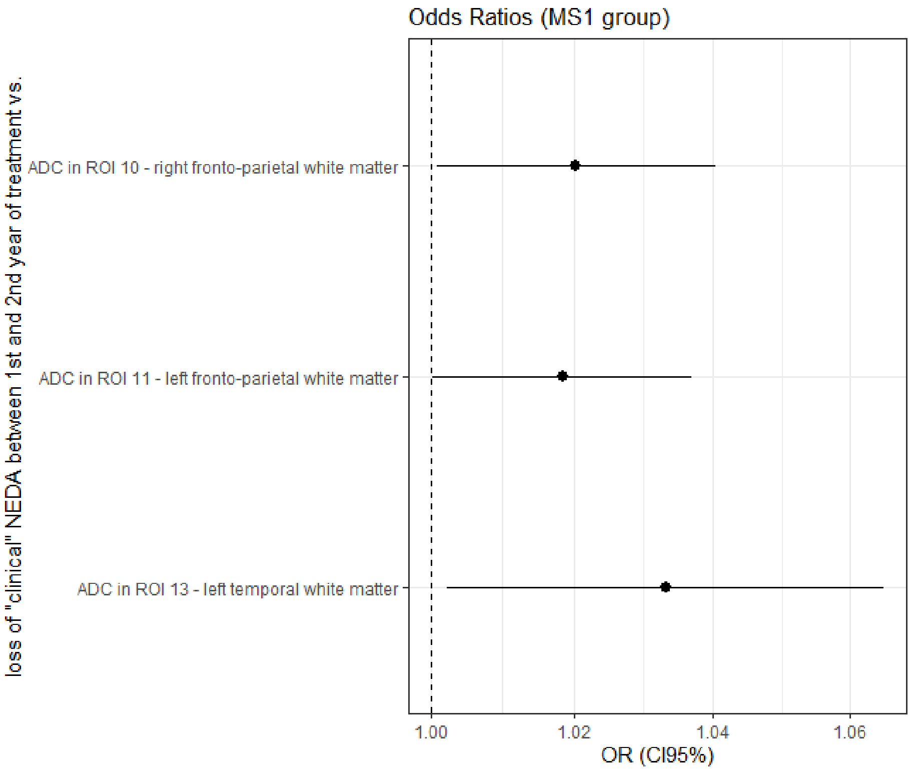
Odds Ratios (MS1 group)
loss of "clinical" NEDA between 1st and 2nd year of treatment vs.
ADC in ROI 10 - right fronto-parietal white matter
ADC in ROI 11 - left fronto-parietal white matter
ADC in ROI 13 - left temporal white matter
1.00
1.02
1.04
1.06
OR (CI95%)

d)

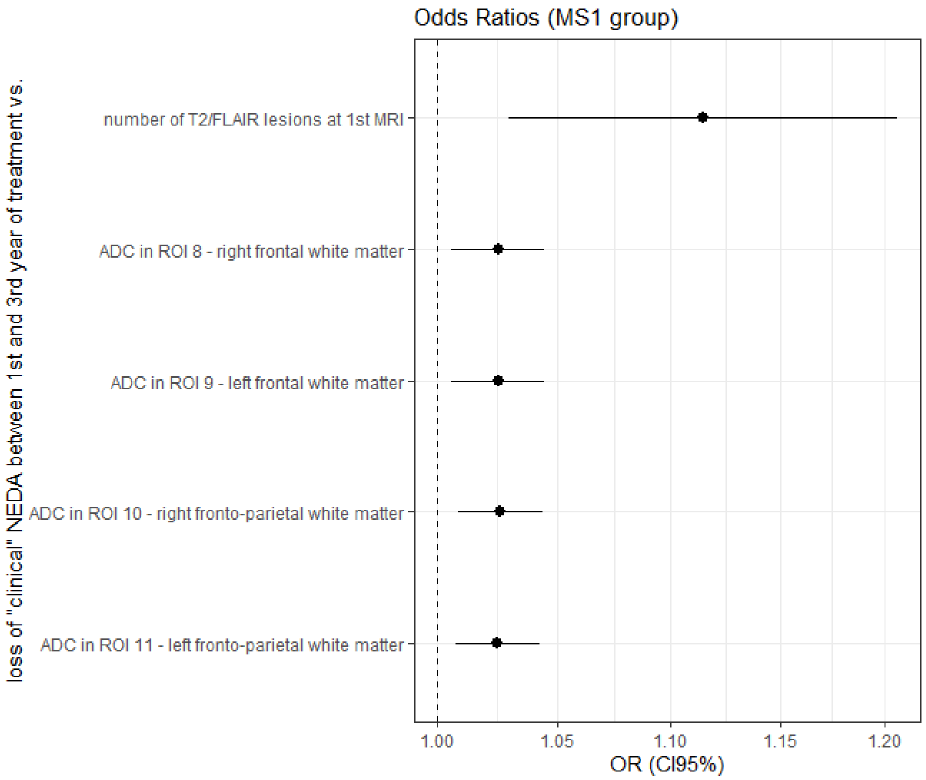

MS – multiple sclerosis, OR – odds ratio, CI – confidence interval, IFN-β – interferon-β, NEDA – no evidence of disease activity, Gd – gadolinium, ADC – apparent diffusion coefficient, ROI – region of interest, ROI 8, 9 – frontal white matter regions right and left, respectively, ROI 10, 11 – frontoparietal white matter at the convexity right and left, respectively , ROI 12, 13 – right and left temporal white matter, respectively, T2 – T2-weighted images, MRI sequence, FLAIR - fluid attenuation inversion recovery, MRI sequence

Suppl. Fig. S2. Receiver operating characteristic (ROC) curves for statistically significant predictors of disease activity during IFN-β treatment in the MS0 group with the 1st MRI before treatment (a, b) and in the MS1 group (c, d, e): (a) ADC in ROI 2 – in the left cerebellar white matter in prognosis of NEDA loss until the 3rd year of treatment in the MS0 group, (b) ADC in ROI 3 – in the pontine white matter in prognosis of "MRI" NEDA loss until the 1st year of treatment in the MS0 group, (c, d, e) – ADC in ROIs 8-13 (both frontal, frontoparietal and temporal white matter) in prediction of the following outcomes – loss of NEDA until the 3rd year of treatment (c), loss of "clinical" NEDA between the 1st and 2nd (d) and between the 1st and 3rd years of treatment (e) in the MS1 group. Moreover, the number of T2/FLAIR lesions contributed to prognosis of the latter endpoint. The best accuracy (≥80%) was achieved in prediction of relapse occurrence between the 1st and 2nd year of treatment (d) for all factors and in prediction of relapses between the 1st and 3rd year of treatment for the ADC value from ROI 10 – in the right frontoparietal white matter (e). Details are depicted in Table 4.

a)

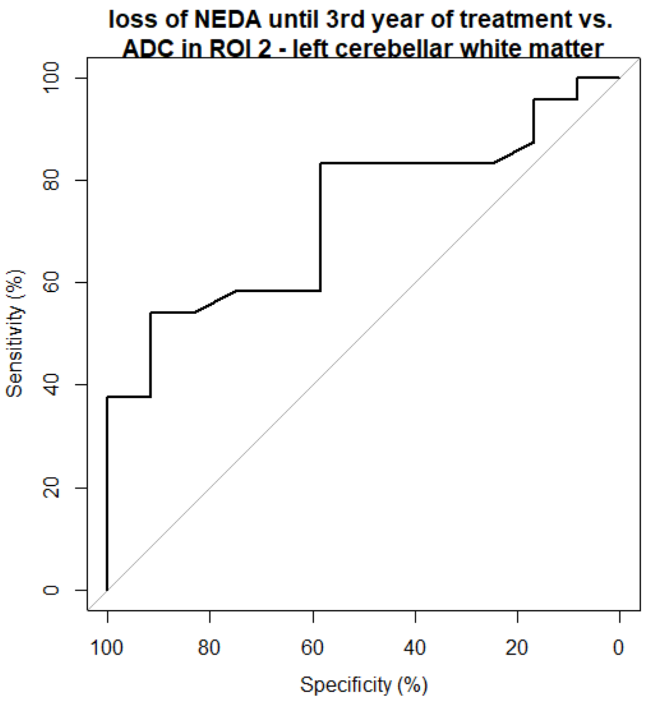

b)

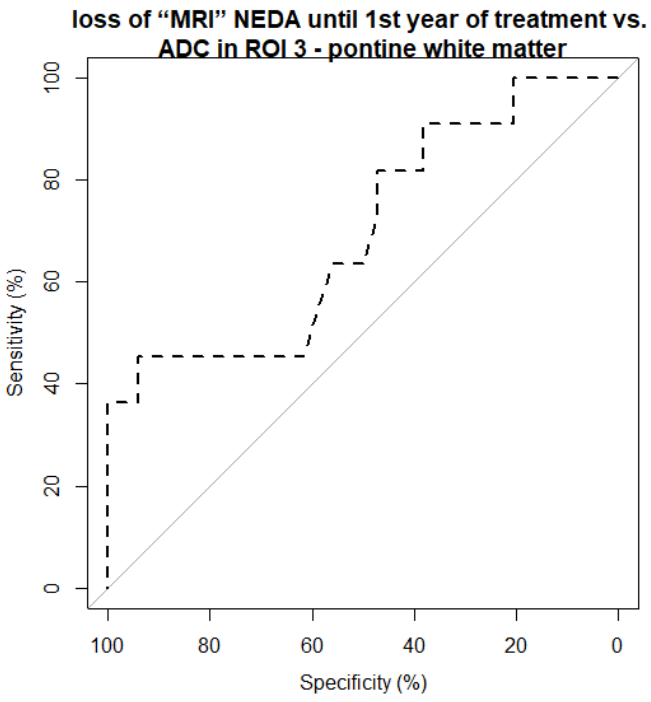

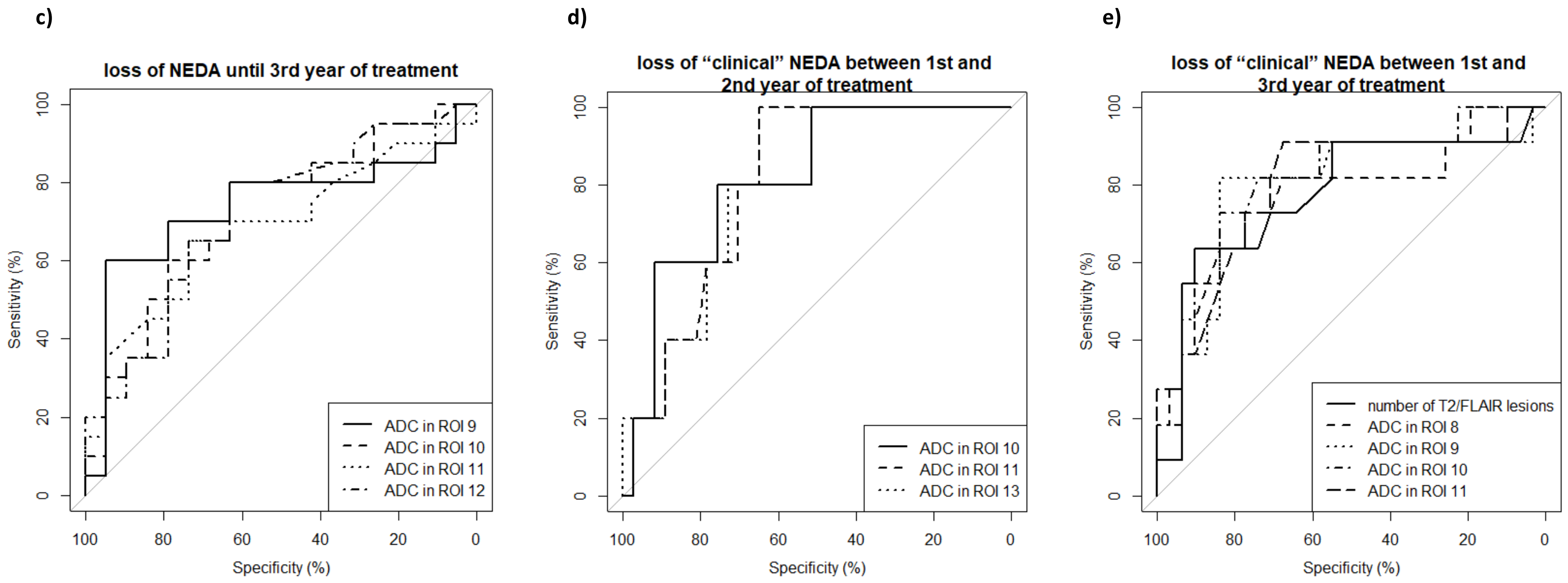

ROC - Receiver Operating Characteristic, MS – multiple sclerosis, IFN-β – interferon-β, NEDA – no evidence of disease activity, ADC – apparent diffusion coefficient, ROI – region of interest, N/A – not applicable, ROI 8, 9 – frontal white matter regions right and left, respectively, ROI 10, 11 – frontoparietal white matter at the convexity right and left, respectively, ROI 12, 13 – right and left temporal white matter, T2 – T2-weighted images, MRI sequence, FLAIR - fluid attenuation inversion recovery, MRI sequence

Suppl. Fig. S3. Cox regression analysis of the associations between the most crucial demographic, clinical and MRI risk factors of time to loss of NEDA and its components (model 3). In the MS0 group, presence of the Gd-enhancing lesion at baseline MRI increased by almost 3 times the risk of reaching loss of NEDA and almost 5 times the risk of reaching loss of "MRI" NEDA (**a**). In the MS1 group ADC values in supratentorial areas obtained at MRI after 1 year of treatment increased the risk of loss of NEDA and its clinical component by about 1%, whereas the number of T2/FLAIR lesions at the 1st MRI increased the risk of reaching "clinical" NEDA loss up to 6% (**b**). Details are described in Table 5.

a)

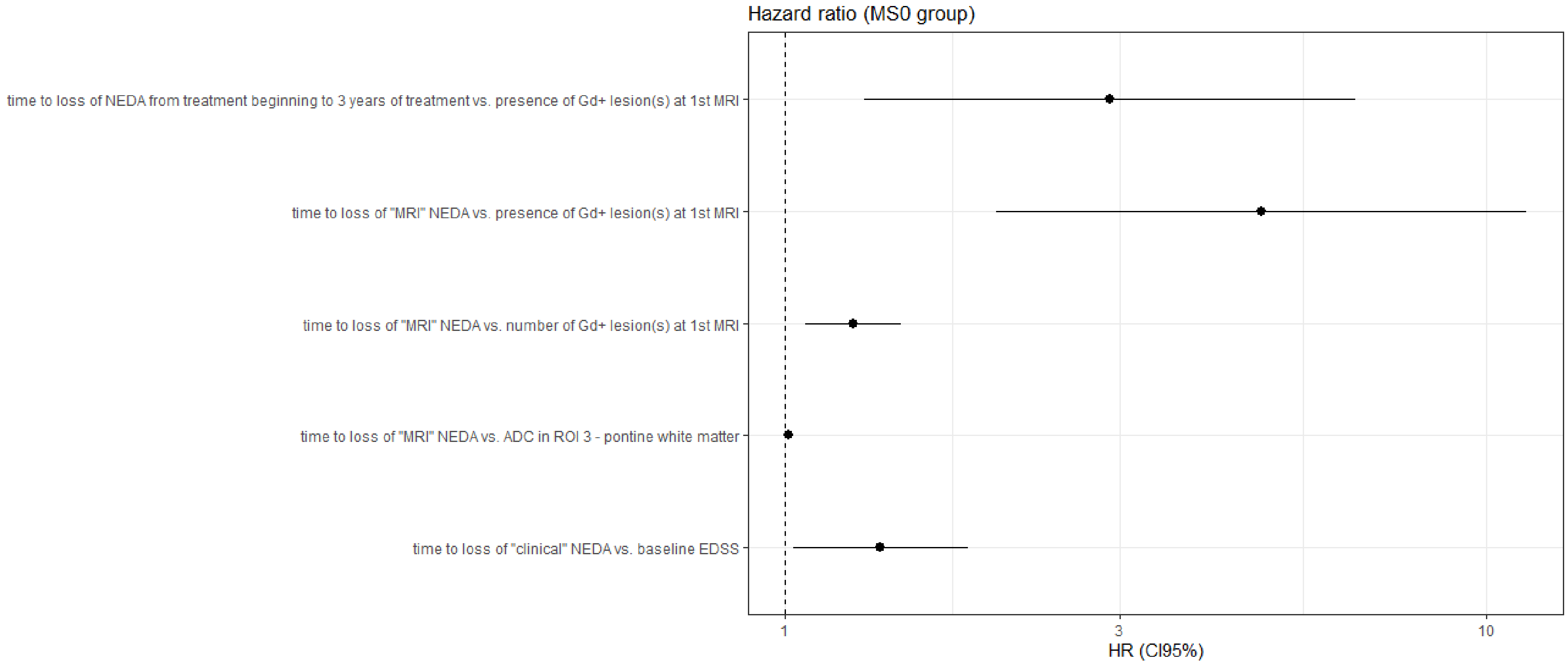

b)

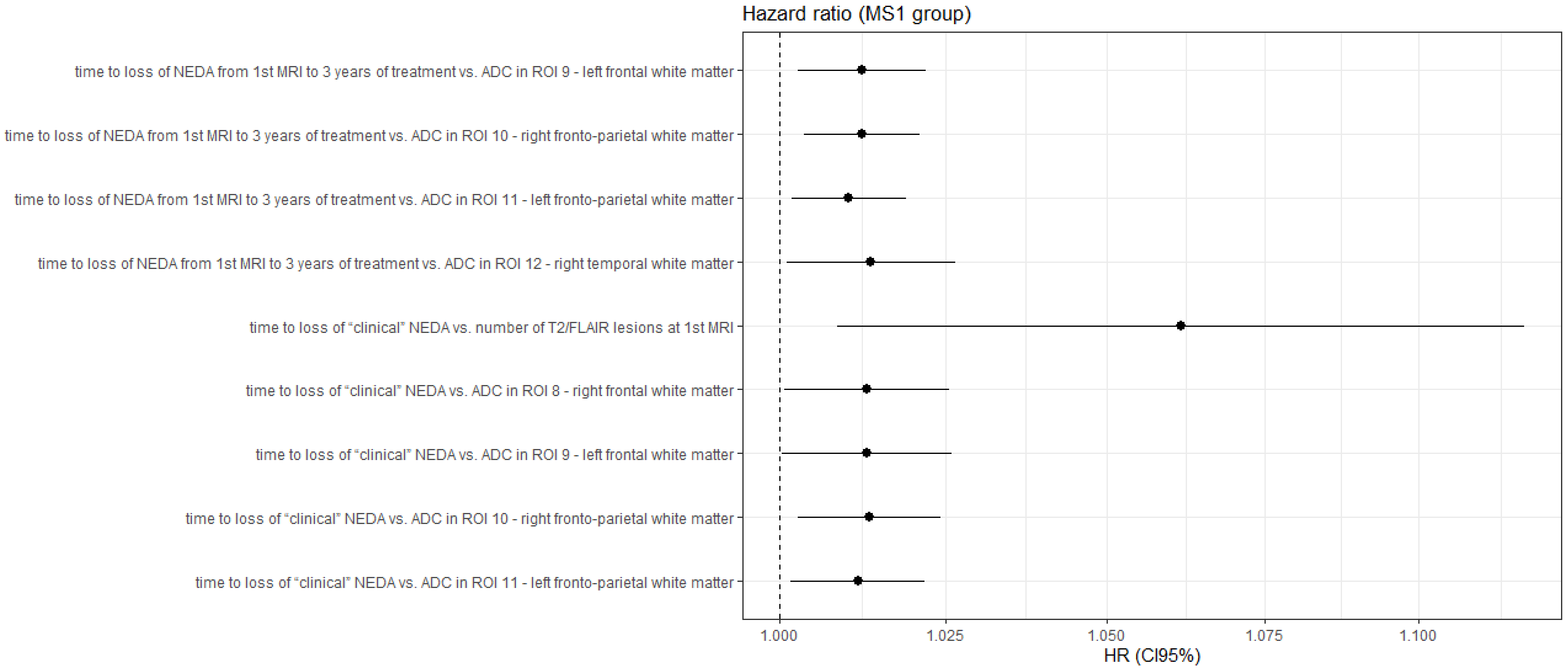

MS – multiple sclerosis, HR – hazard ratio, EDSS – expanded disability status scale, NEDA – no evidence of disease activity, Gd – gadolinium, Gd+ - gadolinium-enhancing, ADC – apparent diffusion coefficient, ROI – region of interest, ROI 3 - pons ,ROI 8, 9 – frontal white matter regions right and left, respectively, ROI 10, 11 – frontoparietal white matter at the convexity right and left, respectively, ROI 12, 13 – temporal white matter right and left, respectively, T2 – T2-weighted images, MRI sequence, FLAIR - fluid attenuation inversion recovery, MRI sequence, FU – follow-up
